# Complete Transposition of Viscera

**Published:** 1918-11

**Authors:** 


**Complete Transposition of Viscera.** Colard and Couturier, Arch. Med. Belges, Feb., describe the demonstration, largely by X-rays, of two cases in soldiers. (Hats off to the Belgian profession for maintaining a medical journal under their present handicap).

